# Intelligent Soft Computing on Forex: Exchange Rates Forecasting with Hybrid Radial Basis Neural Network

**DOI:** 10.1155/2016/3460293

**Published:** 2016-02-08

**Authors:** Lukas Falat, Dusan Marcek, Maria Durisova

**Affiliations:** ^1^Faculty of Management Science and Informatics, University of Zilina, Univerzitna 8215/1, 010 26 Zilina, Slovakia; ^2^Faculty of Economics, VSB-Technical University of Ostrava, Sokolska Trida 33, 701 21 Ostrava 1, Czech Republic

## Abstract

This paper deals with application of quantitative soft computing prediction models into financial area as reliable and accurate prediction models can be very helpful in management decision-making process. The authors suggest a new hybrid neural network which is a combination of the standard RBF neural network, a genetic algorithm, and a moving average. The moving average is supposed to enhance the outputs of the network using the error part of the original neural network. Authors test the suggested model on high-frequency time series data of USD/CAD and examine the ability to forecast exchange rate values for the horizon of one day. To determine the forecasting efficiency, they perform a comparative statistical out-of-sample analysis of the tested model with autoregressive models and the standard neural network. They also incorporate genetic algorithm as an optimizing technique for adapting parameters of ANN which is then compared with standard backpropagation and backpropagation combined with *K*-means clustering algorithm. Finally, the authors find out that their suggested hybrid neural network is able to produce more accurate forecasts than the standard models and can be helpful in eliminating the risk of making the bad decision in decision-making process.

## 1. Introduction

Techniques of artificial intelligence and machine learning started to apply in time series forecasting. One of the reasons was the study of Bollerslev [1], where he proved the existence of nonlinearity in financial data. First models of machine learning applied into time series forecasting were artificial neural networks (ANNs) [2]. This was due to the fact that the artificial neural network is a universal functional black-box approximator of nonlinear type [3–5] that is especially helpful in modeling of nonlinear processes having a priori unknown functional relations or system of relations is very complex to describe [6] and they are even able to model chaotic time series [7]. They can be used for nonlinear modeling without knowing the relations between input and output variables. Thanks to this, ANNs have been widely used to perform tasks like pattern recognition, classification, or financial predictions [8–11]. Following the theoretical knowledge of perceptron neural network published by McCulloch and Pitts [12] and Minsky and Papert [13], nowadays, it is mainly radial basis function (RBF) network [14, 15] that has been used as it showed to be better approximator than the basic perceptron network [16–18].

In this work we extend the standard RBF model by using moving average for modeling the errors of RBF network. We chose the RBF neural network for our exchange rates forecasting experiment because according to some studies [19] ANNs have the biggest potential in predicting financial time series. In addition, Hill et al. [20] showed that ANNs work best in connection with high-frequentional financial data. Moreover, we will combine the standard ANN with EC technique called genetic algorithms. As, according to some scientists [21], the use of technical analysis tools can lead to efficient profitability on the market, we decided to combine our customized RBF network with moving averages [22]. We will use the simple moving average to model the error part of the RBF network as we supposed it could enhance the prediction outputs of the model.

Applying the prediction analysis, the forecasting ability of this nonlinear model will be compared and contrasted with a standard neural network and an autoregressive (AR) model with GARCH errors to determine the best model parameters for this currency pair forecasting problem. We will provide out-of-sample evidence since it focuses directly on predictability as it is important to avoid in-sample overfitting for this type of nonlinear models [23].

The soft computing application we suggest is novel in two ways; we use the standard neural network hybridized with simple moving averages to form a whole new hybrid model. Except for the standard algorithm for training the neural network, we also use other (advanced) technicques.

## 2. Suggested Hybrid-RBF Neural Network Combined with Moving Average

Hybrid models have become popular in the field of financial forecasting in recent years. Since studies from Yang [24] or Clemen [25] theoretically proved that a combination of multiple models can produce better results, we will also use the combined model of customized RBF neural network (supplemented by genetic algorithms for weights adaptation) and simple moving average tool for modeling the error part of the RBF. We eliminate the error of the neural network by modeling the residuals of RBF.

Let *F* be a function defined as* F*: *x*_*t*_ ∈ *ℜ* ^*k*^ → *y*_*t*_ ∈ *ℜ* ^1^ which is a representation assigning one value *y*_*t*_ to *n*-dimensional input in a given time period *t*. Let *G* be a restriction of *F* defined as *G*(*x*_*t*_, *w*_*t*_, *v*_*t*_, *s*) : *x*_*t*_ ∈ *ℜ*_train_^*k*^ → *y*_*t*_ ∈ *ℜ*_train_^1^, where *ℜ*_train_ is a complement of *ℜ*_val_ to *ℜ*. Then, the hybrid neural network model of RBF(*x*, *w*, *v*, *s*) and SMA(*q*) is defined as

(1) $$\begin{array}{c} G\left(\;x_{t},w_{t},v_{t},s\;\right)=\psi_{2}\overset{s}{\underset{j=1}{\sum }}\left(\;v_{j}o_{j}\;\right)+\varepsilon_{t}^{RBF}, \end{array}$$

where

(2) $$\begin{array}{c} o_{j}=\psi_{1}\left[\;\phi \left(\;x,w^{j}\;\right)\;\right],\;j=1,2,\ldots ,s \end{array}$$

with

(3) εtRBF=et+ut,ut≈iid0,1,et=∑i=1qθiεt−iRBF,∑i=1qθi=1,Ewt=∑xt,yt∈RtrainkGxt,wt,vt,s−yt2,Ewt⟶min,εtRBF-SMA=Gxt,wt,vt,s−y.

The necessary condition is that the model must be adapted to approximate the unknown function *F*; that is, the model must fulfill the condition that the difference between estimated output produced by the model and the original value is minimal.

*x*, *w*, *v*, *s* denote the ANN parameters; *x* is the input vector of the dimension *n*; *w* and *v* are the parameters of the network (also called synapses or weights) and are used for the interconnection of the neural network. The input vector *x*_*t*_^*T*^ = (*x*_1*t*_, *x*_2*t*_,…, *x*_*kt*_) forms the input layer of the network; *w*_*j*_ are weights going from the input layer to the hidden layer that is formed by *s* hidden neurons. In the RBF network, the radial basis function of Gaussian type instead of a sigmoid function is used for activating neurons in hidden layer of a perceptron network. The Gaussian function for activating neurons is for *j*th hidden neuron defined as *ψ*_1_(*u* ^*j*^) = *e* ^−*u*^*j*^/2*σ*_*j*_^2^^ = *e* ^−||*x* − *w*^*j*^ | |^2^/2*σ*_*j*_^2^^, *j* = 1,2,…, *s*, where *σ*_*j*_^2^ is the variance of *j*th neuron and *u* is the potential of the neuron. Furthermore, *v*_*j*_, *v*_*j*_ ∈ *R* are weights between *j*th hidden neuron and the output layer that is represented by just one neuron (the network output). Activated neurons are weighted by weight vector *v*_*t*_^*T*^ = (*v*_1*t*_, *v*_2*t*_,…, *v*_*st*_) in order to get the output of the network counted as *y* = *ψ*_2_(*u*_*t*_) = ∑_*j*=1_^*s*^ *v*_*j*_ *o*_*j*_.

## 3. Methodology

The neural network we used for this research was RBF which is one of the most frequently used networks for regression. RBF has been widely used to capture a variety of nonlinear patterns (see [26]) thanks to their universal approximation properties (see [27]).

In order to optimize the outputs of the network and to maximize the accuracy of the forecasts we had to optimize parameters of ANN. The most popular method for learning in multilayer networks is called backpropagation. It was first invented by Bryson and Ho [28]. But there are some drawbacks to backpropagation. One of them is the “scaling problem.” Backpropagation works well on simple training problems. However, as the problem complexity increases (due to increased dimensionality and/or greater complexity of the data), the performance of backpropagation falls off rapidly [29]. Furthermore, the convergence of this algorithm is slow and it generally converges to any local minimum on the error surface, since stochastic gradient descent exists on a surface which is not flat. So the gradient method does not guarantee to find optimal values of parameters and imprisonment in local minimum is quite possible.

As genetic algorithms have become a popular optimization tool in various areas, in our implementation of ANN, backpropagation will be substituted by the GA as an alternative learning technique in the process of weights adaptation. Genetic algorithms (GA), which are EC algorithms for optimization and machine learning, are stochastic search techniques that guide a population of solutions towards an optimum using the principles of evolution and natural genetics [30]. Adopted from biological systems, genetic algorithms are based loosely on several features of biological evolution [31]. In order to work properly, they require five components [32], that is, a way of encoding solutions to the problem on chromosomes, an evaluation function which returns a rating for each chromosome given to it, a way of initializing the population of chromosomes, operators that may be applied to parents when they reproduce to alter their genetic composition, parameter settings for the algorithm, the operators, and so forth. GA are also characterized by basic genetic operators which include reproduction, crossover, and mutation [33]. Given these genetic operators and five components stated above, a genetic algorithm operates according to the following steps stated in [29]. When the components of the GA are chosen appropriately, the reproduction process will continually generate better children from good parents; the algorithm can produce populations of better and better individuals, converging finally on results close to a global optimum. Additionally, GA can efficiently search large and complex (i.e., possessing many local optima) spaces to find nearly global optima [29]. Also, GA should not have the same problem with scaling as backpropagation. One reason for this is that it generally improves the current best candidate monotonically. It does this by keeping the current best individual as part of their population while they search for better candidates.

In addition, as Kohonen [34] demonstrated that nonhierarchical clustering algorithms used with artificial neural networks can cause better results of ANN, unsupervised learning technique will be used together with RBF in order to find out whether this combination can produce the effective improvement of this network in the domain of financial time series. We will combine RBF with the standard unsupervised technique called *K*-means (see [35]). *K*-means algorithm, which belongs to a group of unsupervised learning methods, is a nonhierarchical exclusive clustering method based on the relocation principle. The most common type of characteristic function is location clustering. And the most common distance function is Euclidean.

The *K*-means will be used in the phase of nonrandom initialization of weight vector *w* performed before the phase of network learning. In many cases it is not necessary to interpolate the output value by radial functions, it is quite sufficient to use one function for a set of data (cluster), whose center is considered to be a center of activation function of a neuron. The values of centroids will be used as initialization values of weight vector *w*. Weights should be located near the global minimum of the error function (1) and the lower number of epochs is supposed to be used for network training. The reason why we decided to use *K*-means is that it is quite simple to implement and in addition to that, in the domain of nonextreme values, it is relatively efficient algorithm. In our experiments, the adaptive version of *K*-means will be used which is defined as follows:

(1) random initialization of centroids in the dimension of input vector,
(2) introduction of input vector *x*_*i*_,
(3) determination of the nearest from all centroids to a given input,
(4) adaptation of the coordinates of the centroid according to the rule as follows: *c*_*j*′_ = *c*_*j*′_^*∗*^ + *η*(*x*_*i*_ − *c*_*j*′_), where *j*′ is the nearest cluster to the introduced input and *η* is a learning rate parameter,
(5) termination of the algorithm if all inputs were processed or the coordinates of the cluster are not changing anymore.

## 4. Empirical Research

We chose forex market for our experiments. Our experiment focuses on time series of daily close price of USD/CAD (the data were downloaded from a website http://www.global-view.com/forex-trading-tools/forex-history/) (Canadian dollar versus US dollar), one of major currency pairs, covering a historical period from October 31, 2008, to October 31, 2012 (*n* = 1044 daily observations). Due to validation of a model, data were divided into two parts (Figure 7). The first part included 912 observations (from 10/31/2008 to 4/30/2012) and was used for the model training. The second part of data (5/1/2012 to 10/31/2012), counting 132 observations, was used for model validation by making one-day-ahead ex-post forecast. These observations include new data which have not been incorporated into model estimation (parameters of the model were not changing anymore in this phase). The reason for this procedure is the fact that an ANN can become so specialized for the training set that loses flexibility, hence the accuracy in the test set.

We used our own application of RBF neural network implemented in JAVA with one hidden layer according to Cybenko [36]; the feedforward network with one hidden layer is able to approximate any continuous function. For the hidden layer, the radial basis function was used as an activation function as it has been showed that it provides better accuracy than the perceptron network. We estimated part of the RBF model with several adapting algorithms: RBF implemented with a backpropagation algorithm, a genetic algorithm, and combination of *K*-means and backpropagation. As for the backpropagation learning, the learning rate was set to 0.001 to avoid the easy imprisonment in local minimum. The number of epochs for each experiment with backpropagation was set to 5000 as this showed to be a good number for backpropagation convergence. The final results were taken from the best of 5000 epochs and not from the last epoch in order to avoid overfitting of the neural network. *K*-means was used instead of random initialization of weights before they were adapted by backpropagation. Coordinances of clusters were initiated as coordinances of randomly chosen input vector. *K*-means cycle was repeated 5000 times and the learning rate for cluster adaptation was set to 0.001. The number of clusters was set to the number of hidden neurons.

For GA algorithm the following was needed: a method of encoding chromosomes, the fitness function used to calculate the fitness values of chromosomes, the population size, initial population, maximum number of generations, selection method, crossover function, and mutation method. Our implementation of the genetic algorithm we used for weight adaptation is as follows. The chromosome length was set according to the formula: *D∗s* + *s*, where *s* is the number of hidden neurons and *D* is the dimension of the input vector. A specific gene of a chromosome was a float value and represented a specific weight in the neural network. The whole chromosome represented weights of the whole neural network. The fitting function for evaluating the chromosomes was the mean square error function (MSE). The chromosome (individual) with the best MSE was automatically transferred into the next generation. The other individuals of the next generation were chosen as follows: by tournament selection 100 individuals were randomly chosen from the population. The fittest of them was then chosen as a parent. The second parent was chosen in the same way. The new individual was then created by crossover operation. If the generated value from <0, 1 was lower than 0.5 the weight of the first parent at the specific position was assigned to the new individual. Otherwise, the new individual received the weight of the second parent. The mutation rate was set to 0.01. If performed, the specific gene (weight) of a chromosome was changed to a random value. The size of the population and the number of generations for the genetic algorithm were set accordingly to the settings of backpropagation. Based on some experiments, we used the size of the population that equaled 1000 and the number of generations was set to 10.

When the best configuration of the RBF network was found, the RBF error was then modelled in order to minimize the total error of the model. Using moving average, the forecast of the future error of the RBF was counted as an average of last network errors. We used only simple moving average: the weights of the previous network errors had the same weight. To find out the optimal number of the parameters of moving average tool, we used various numbers of previous errors for counting the future (average) value of RBF error.

The numerical characteristic for assessing models called mean squared error (MSE) was used:

(4) $$\begin{array}{c} MSE=H^{-1}\overset{H}{\underset{h=1}{\sum }}{\left(\;{\overset{\wedge }{Y}}_{n-H+h}-Y_{n-H+h}\;\right)}^{2}, \end{array}$$

where *h* is the forecasting horizon and *H* is the total number of predictions for the horizon *h* over the forecast period.

In order to make a comparison with standard statistical models, we also performed the empirical Box-Jenkins analysis [37] in order to compare our suggested model with standard statistical model (for details of Box-Jenkins analysis see the appendix). Box-Jenkins analysis focused on the original and differentiated series of daily observations of USD/CAD currency pair covering a historical period from October 31, 2008, to October 31, 2012. The data, as stated above, was downloaded from the following website: http://www.global-view.com/forex-trading-tools/forex-history/. The best results for out-of-sample prediction were achieved with EGARCH(1,1, 1) model with Gaussian error distribution. Therefore, this model was compared with the neural network models and our suggested model. The volatility of this model is defined as follows:

(5) log⁡ht=−0.172109+0.117148εt−1ht+0.037398εt−1ht+0.992135log⁡ht−1.

## 5. Results and Discussion

The reason why the prediction qualities were applied on the validation set (ex-ante predictions) was the fact that an ANN can become so specialized for the training set that could lose accuracy in the test set. Therefore, the estimation of all models was only based on 912 observations, in order to make further comparisons with the predictions of the 132 remaining observations. In this paper, we only used one-step-ahead forecast: that is, horizon of predictions was equal to one day. In order to eliminate deformation of our results by a single replication we used a procedure applied in Heider et al. [38]; that is, experiment for every model configuration was performed twelve times, the best and worst results were eliminated, and from the rest the mean and standard deviations were counted. The result of a given model is from the best neuron configuration (in every model we tested number of hidden neurons from 3 to 10 to find the best output results of the network).

**Figure 1:**
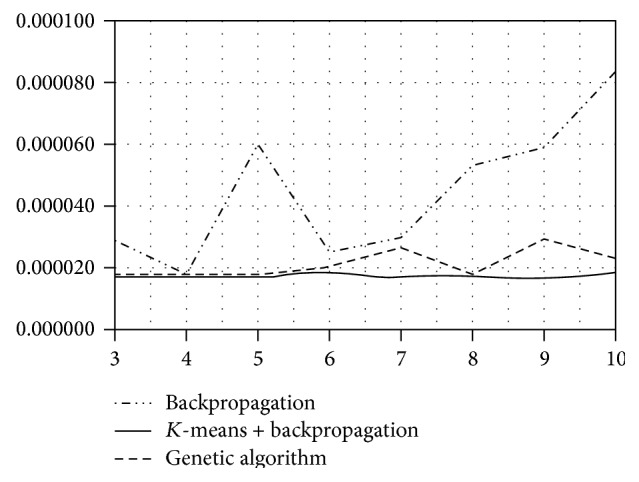
Predictive accuracy of the standard RBF network, AR(1) input.

In Table 1 (RBF network, one autoregressive input), we see that network with BP achieved the best results when having 4 neurons in the hidden layer (see also Figure 1). On the other hand, the advanced methods for network learning (*K*-means + BP, GA) achieved the best results with 4 (GA), respectively, 9 neurons (*K*-means + BP). However, when using these advanced methods the number of hidden neurons seemed to not play an important role as the results were comparable. Following from that one can deduce that for remembering the relationships in this time series it is enough to use smaller number of hidden neurons (three or four). When looking at the results of the standard BP, the reason for increasing the error with the higher number of neurons is the fact that the more of the neurons the longer time for the weights adaptions of the network.

Also, the standard BP was the great weakness of the neural network. The convergence is really slow and it generally converges to any local minimum on the error surface, since stochastic gradient descent exists on a surface which is not flat. So the gradient method does not guarantee to find optimal values of parameters and imprisonment in local minimum is quite possible. Another drawback to backpropagation is the “scaling problem.” Backpropagation works well on simple training problems. However, as the problem complexity increases (due to increased dimensionality and/or greater complexity of the data), the performance of backpropagation falls off rapidly.

**Figure 2:**
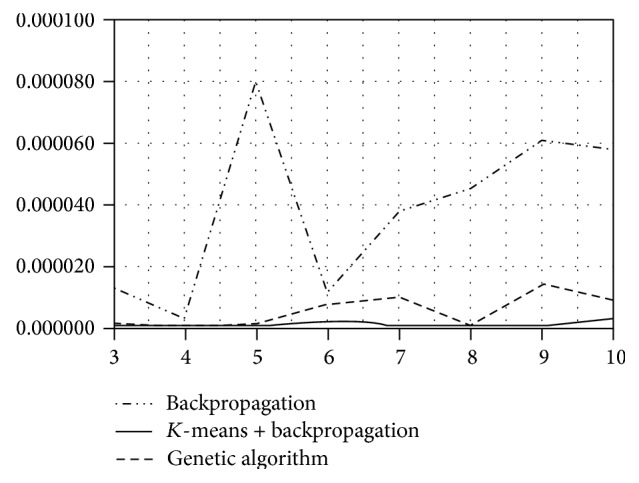
Standard deviation of the standard RBF network, AR(1) input.

Due to these disadvantages of BP, we tested other methods for network adaptation. No surprise that the RBF network combined with *K*-means or GA for weights adaptation provided significantly better results than the original RBF (see Table 1). Moreover, besides lower mean MSE, another advantage of using genetic algorithm or *K*-means upgrade is the consistency of predictions, that is, standard deviation of performed experiments at the same network configuration (see Figure 2). The standard deviation of these methods is uncomparably lower than the standard deviation when using the standard backpropagation (see Table 1 and Figure 2).

The biggest strength of *K*-means is the speed of convergence of the network. Without *K*-means, it took considerably longer time to achieve the minimum. However, when the *K*-means was used to set the weights of the network before backpropagation, the time for reaching the minimum was much shorter. The advantage of this combination is that lower number of epochs is supposed to be used for network training. Moreover, *K*-means is quite simple to implement. However, one must bear in mind that *K*-means is a relatively efficient algorithm only in the domain of nonextreme values.

We tested also GA in weights adaptation and we found out that the convergence is also considerably faster than at backpropagation and therefore it is no surprise that sometimes the network converged only after 5 generations. In addition to that, genetic algorithm does not have the same problem with scaling as backpropagation. One reason for this is that it generally improves the current best candidate monotonically. It does this by keeping the current best individual as part of their population while they search for better candidates. Genetic algorithms are generally not bothered by local minima. Also, genetic algorithms are especially capable of handling problems in which the objective function is discontinuous or nondifferentiable, nonconvex, multimodal, or noisy. Since the algorithms operate on a population instead of a single point in the search space, they climb many peaks in parallel and therefore reduce the probability of finding local minima. In other words, a key concept for genetic algorithms is the schemata. A schema is a subset of the fields of a chromosome set to particular values with the other fields left to vary. Therefore, as originally observed in Holland [31], the power of genetic algorithms lies in their ability to implicitly evaluate large numbers of schemata simultaneously and to combine smaller schemata into larger schemata [29]. The disadvantage of using genetic algorithms in the neural network is the fact that it demands quite a lot of parameters to set it up correctly (population size, mutation rate, crossover function, crossover rate, tournament size, fitness function, etc.).

When comparing weights adaptation via GA and *K*-means plus backpropagation, the results are almost the same. Even though *K*-means provided better results compared to GA, the differences are not very large. However, GA has a bigger potential to perform even better forecasts as there are more parameters needed to be optimized. Backpropagation, even though it is used with *K*-means, seemed to reach its global minimum even with the higher number of epochs (we tested backpropagation up to 10000 cycles) and the results were almost the same.

For assessing our new hybrid neural network model we used the same strategy as for the standard ANN. For the value of parameter of the moving average, we tested the values from one to one hundred and we experimentally found out the best value for the tested data (for the majority of testing procedures the optimal value of moving average parameter was 44). Finally, just like for the standard RBF, from the best ten out of twelve experiments, the mean and standard deviations of the best results of suggested hybrid (having the optimal value of MA parameter) were counted. For every number of hidden neurons tested, the results are stated in Table 1 which contains the results of out-of-sample predictions provided by the different models and optimization techniques, respectively. The illustrated results from one testing procedure are shown in Table 2 (it is important to note that the final results presented in Table 1 are made as the mean and standard deviation of ten procedures like the one in Table 2).

**Figure 3:**
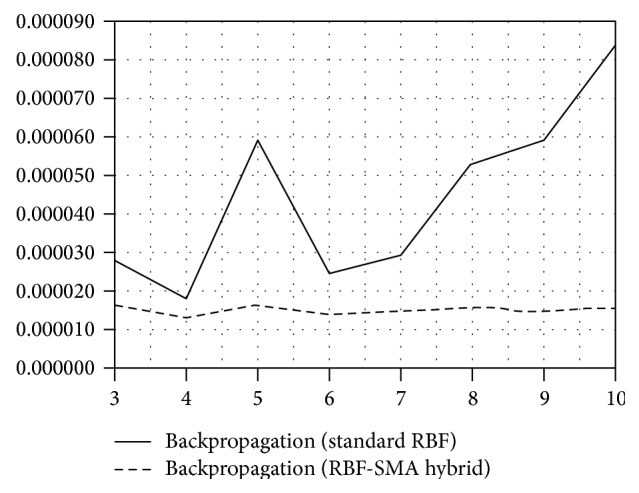
Predictive accuracy of standard RBF model and RBF-MA hybrid model (BP algorithm).

**Figure 4:**
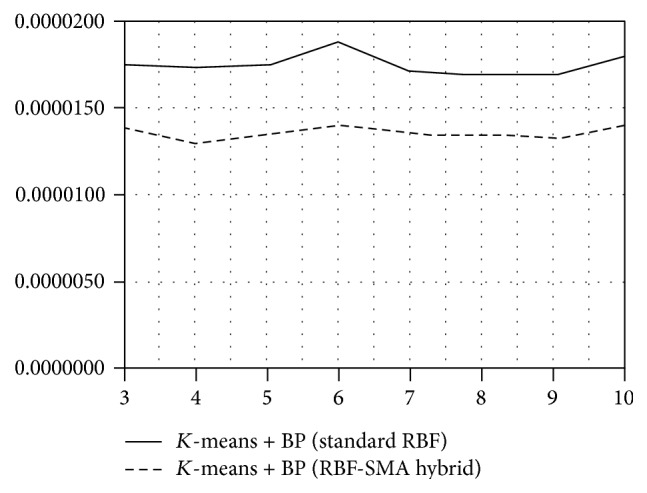
Predictive accuracy of standard RBF model and RBF-MA hybrid model (*K*-means + BP).

**Figure 5:**
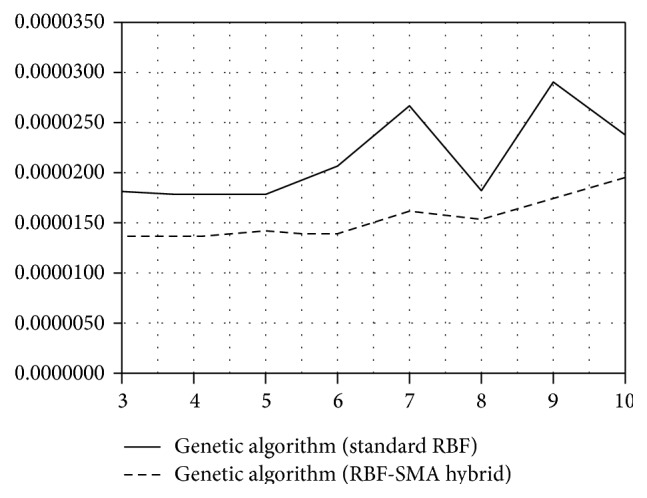
Predictive accuracy of standard RBF model and RBF-MA hybrid model (genetic algorithm).

**Figure 7:**
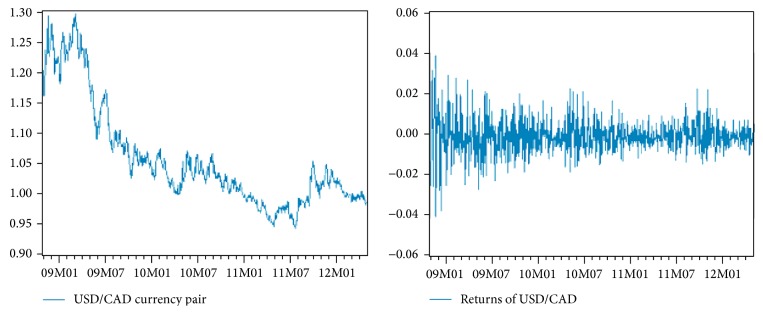
USD/CAD (original series and differences of the original series).

We also performed the predictive comparison with standard RBF network as well as the statistical ARIMA and GARCH model in order to show the prediction power of our suggested model.  Table 4 states the final results of the numerical comparison of tested models. The standard RBF provided the best outputs when combined with *K*-means and backpropagation algorithms. The error of prediction at this network was a little bit lower compared to statistical model; however, these two models provided almost the same results. Nonetheless, the suggested hybrid neural network model provided much better forecasts compared to these two models. Comparing the numerical (see Table 3) as well as graphical results (see Figures 3, 4, and 5), the hybrid improved the prediction power of the standard RBF considerably. We can state that the application of our suggested new hybrid neural network model into the domain of exchange rates provides significantly better results than the standard RBF neural network as well as statistical models.

## 6. Conclusion

Quantitative methods are excellent tool in decision-making process as they rely on facts, numbers, and accurate mathematical methods and models. The most used approach, which has been used for many years, is a statistical approach. Statistical methods are verified methods which have been used in forecasting process for many years. As for computing intelligent technologies, they are getting more and more popular nowadays. The main representative neural networks are based on mathematical model of human neuron and therefore it does not have to fulfil any initial assumptions like statistical models. In this paper we tested the predictive power of neural networks in the domain of exchange rates. We suggested a new hybrid network model combined with moving averages. We used USD/CAD data which was later divided into training set and validation due to model checking. We also performed the tests with the statistical model. We also used other algorithms in the neural network training process; we combined RBF with an unsupervised learning method called *K*-means and GA into the RBF. The reason for incorporating other algorithms into the network was that the BP is considered a weakness of the RBF. Both of these upgrades showed to be helpful in the process of creating better forecasts and should be definitely used instead of the standard BP.

By performing experiments we can deduce that the models of ANN are relatively fast, they are able to generalize, and in addition to that it is not necessary to know all the relationships of the system. Thanks to that, ANN modeling is enabled to people who are not able to identify relations between the variables of the model by using Box-Jenkins, GARCH, or any other methodology. Moreover, in this work we also suggested a new hybrid neural network model. The reason for this was to improve the prediction accuracy of our customized standard neural network. As for the prediction results of our hybrid, we performed experiments to find out that our suggested RBF-MA hybrid neural network has a significant predictive superiority over the statistical model as well as standard neural network models. On the validation set the tested hybrid model proved excellent results and according to MSE errors on the validation set, it was by far the best model of all tested models. In our experiments its numerical characteristics always overcame individual models (ANN, statistical model); the improvements ranged from about 18 per cent to more than 89 per cent. Our hybrid neural network model showed to be a great improvement of the standard RBF neural network as we experimentally clearly proved that for the USD/CAD this hybrid model provided significantly better forecasts than the standard model of the RBF neural network and as the statistical model and hence there was a clear benefit of better one-day-ahead forecasts.

Despite the fact that neural networks and soft computing techniques are a minor approach used in decision process of business forecasting, it is definitely an attractive alternative to traditional statistical models. Moreover, following from our empirical findings for out-of-sample one-step-ahead forecasts, we believe that our suggested hybrid model has also a great potential in the whole domain of financial forecasting as well as other areas of continuous forecasting.

## Figures and Tables

**Figure 6:**
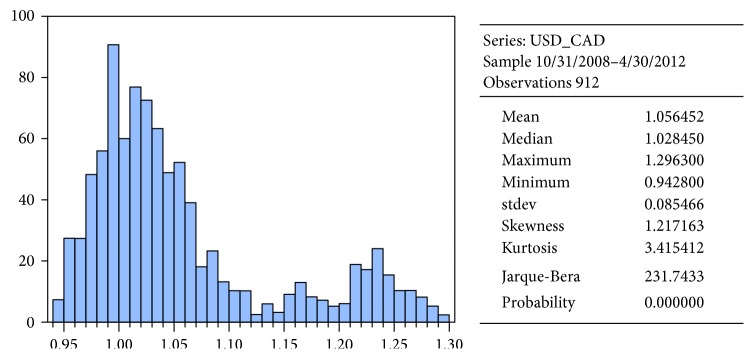
Histogram and statistical characteristics of original series of USD/CAD (training set).

**Figure 8:**
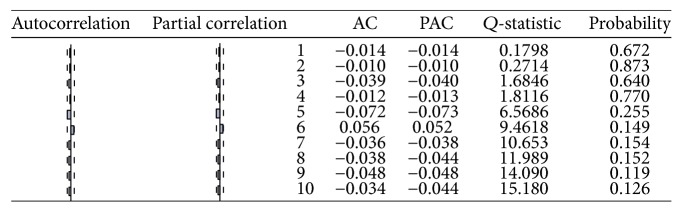
ACF and PACF of USD/CAD returns.

**Figure 9:**
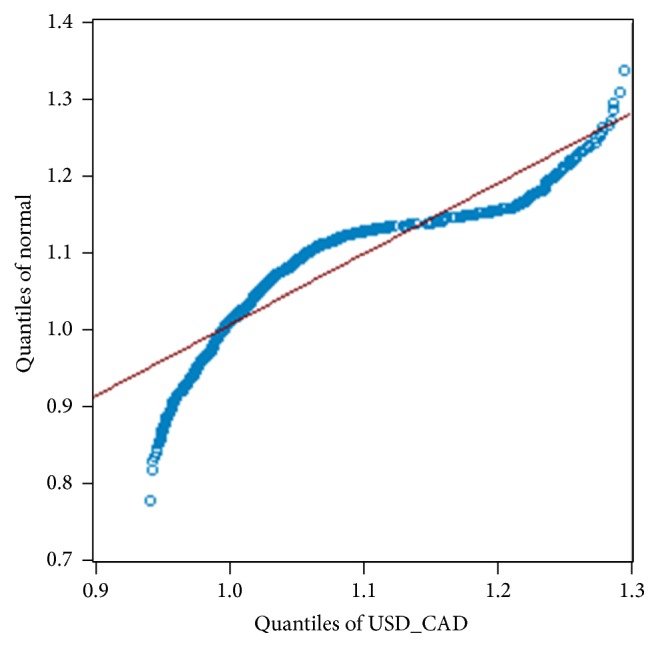
Quantiles of USD/CAD residuals versus the normal distribution quantiles.

**Figure 10:**
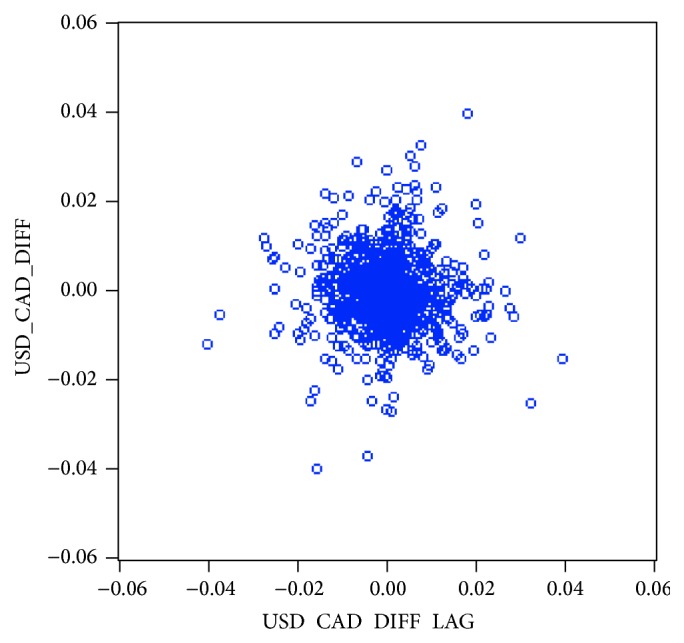
Scatter plot of USD/CAD residuals variations.

**Figure 11:**
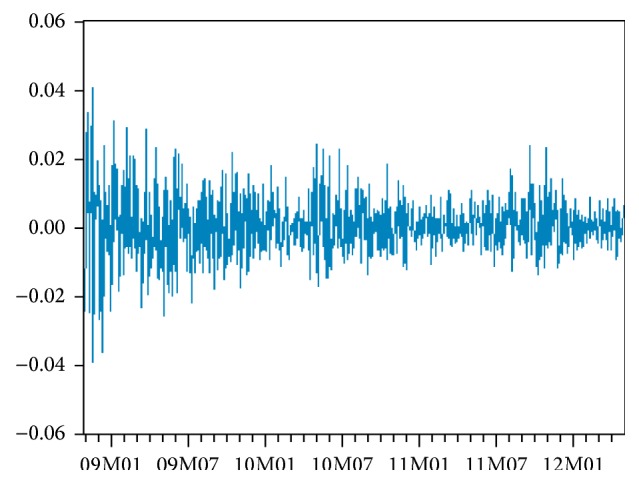
Evolution of residuals.

**Figure 12:**
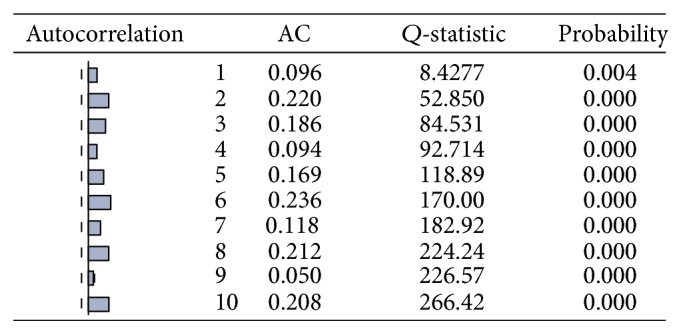
Correlogram of squared residuals.

**Figure 13:**
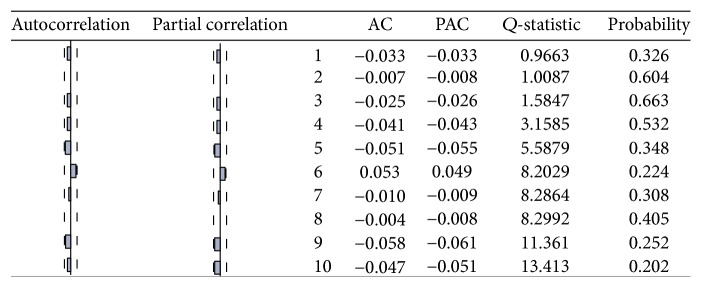
ACF and PACF of AR(0)-GARCH(1,1) GED residuals.

**Table 1:** Predictive qualities of tested models on ex-post (out-of-sample predictions).

Neurons	Network optimization	RBF network		Hybrid neural network	
		MSE	stdev	MSE	stdev
3	Backpropagation	0.0000282628	0.0000129939	0.0000169513	0.0000039062
	*K*-means + backpropagation	0.0000175381	0.0000006224	0.0000137675	0.0000009931
	Genetic algorithm	0.0000180929	0.0000016469	0.0000136146	0.0000003816

4	Backpropagation	0.0000183763	0.0000028765	0.0000136485	0.0000005710
	*K*-means + backpropagation	0.0000173006	0.0000004025	0.0000130549	0.0000003013
	Genetic algorithm	0.0000176860	0.0000006219	0.0000137306	0.0000010974

5	Backpropagation	0.0000299369	0.0000812952	0.0000168334	0.0000069884
	*K*-means + backpropagation	0.0000174326	0.0000007575	0.0000133526	0.0000003885
	Genetic algorithm	0.0000176925	0.0000016246	0.0000141386	0.0000011016

6	Backpropagation	0.0000248756	0.0000105719	0.0000140990	0.0000016518
	*K*-means + backpropagation	0.0000187115	0.0000024836	0.0000140002	0.0000011530
	Genetic algorithm	0.0000205995	0.0000073265	0.0000139753	0.0000010496

7	Backpropagation	0.000029955	0.0000381995	0.0000152401	0.0000018918
	*K*-means + backpropagation	0.0000170959	0.0000002617	0.0000135883	0.0000004315
	Genetic algorithm	0.0000265817	0.0000100553	0.0000160908	0.0000033735

8	Backpropagation	0.0000530843	0.0000462909	0.0000161911	0.0000018501
	*K*-means + backpropagation	0.0000169521	0.0000003200	0.0000133422	0.0000002243
	Genetic algorithm	0.0000181709	0.0000016133	0.0000152679	0.0000030365

9	Backpropagation	0.0000594814	0.0000611668	0.0000156977	0.0000018874
	*K*-means + backpropagation	0.0000168649	0.0000002319	0.0000132936	0.0000003833
	Genetic algorithm	0.0000290958	0.0000136948	0.0000174571	0.0000049429

10	Backpropagation	0.0000842809	0.0000580551	0.0000163252	0.0000019133
	*K*-means + backpropagation	0.0000179805	0.0000029834	0.0000139659	0.0000011918
	Genetic algorithm	0.0000236821	0.0000093964	0.0000193432	0.0000056131

stdev: standard deviation.

**Table 2:** Prediction power of suggested hybrid model (backpropagation, one input).

MA order	MSE
0 (standard RBF)	1.873950157362012*E* − 5
1	3.799165246508804*E* − 5
2	2.615127248199574*E* − 5
3	2.367687507828842*E* − 5
4	2.2783554024814407*E* − 5
5	2.0696629830254916*E* − 5
6	2.005212457137589*E* − 5
7	1.9699739236710283*E* − 5
8	1.899554614297672*E* − 5
9	1.8775515147039734*E* − 5
10	1.887215699290597*E* − 5
11	1.902577681032068*E* − 5
12	1.8665433411401154*E* − 5
13	1.8538236785067435*E* − 5
14	1.8747363637004875*E* − 5
15	1.877614881966081*E* − 5
16	1.885927703360986*E* − 5
17	1.8730311643222403*E* − 5
18	1.865192824881276*E* − 5
19	1.8613846327275632*E* − 5
20	1.8246180732788623*E* − 5
21	1.8304204212300793*E* − 5
22	1.8346810489111456*E* − 5
23	1.8767301251545768*E* − 5
24	1.8221445314293524*E* − 5
25	1.8337288088681414*E* − 5
26	1.8372328768988*E* − 5
27	1.713290433035233*E* − 5
28	1.7387905538550667*E* − 5
29	1.7455556353006092*E* − 5
30	1.76365575048565*E* − 5
31	1.7430582353411663*E* − 5
32	1.7628308525319124*E* − 5
33	1.7295489520120636*E* − 5
34	1.75881015020376*E* − 5
35	1.7826153183101944*E* − 5
36	1.71971230137477*E* − 5
37	1.733159545759489*E* − 5
38	1.6866625811781463*E* − 5
39	1.668511605555683*E* − 5
40	1.686512816301278*E* − 5
41	1.6615393182238008*E* − 5
42	1.674994621240823*E* − 5
43	1.6412484212686543*E* − 5
44	1.3700534874132779*E* − 5
45	1.3759565540757362*E* − 5
46	1.3741032598505082*E* − 5
47	1.3991779046903492*E* − 5
48	1.4203358839945669*E* − 5
49	1.4122256244441945*E* − 5
50	1.428843656456151*E* − 5
51	1.4470021893018167*E* − 5
52	1.4604793623824943*E* − 5
53	1.4639288195772851*E* − 5
54	1.460226532797794*E* − 5
55	1.4861518152409294*E* − 5
56	1.5056461844185913*E* − 5
57	1.5072953046357367*E* − 5
58	1.5151285670421108*E* − 5
59	1.5147537079794747*E* − 5
60	1.5206339461136763*E* − 5
61	1.5288938970910543*E* − 5
62	1.4741449702485418*E* − 5
63	1.447140949098859*E* − 5
64	1.4028230686891647*E* − 5
65	1.4262672084968514*E* − 5
66	1.4502514602499232*E* − 5
67	1.4728307701510205*E* − 5
68	1.4835695748235584*E* − 5
69	1.4287605837827516*E* − 5
70	1.4434608041518276*E* − 5
71	1.4617126171765766*E* − 5
72	1.4774475331564346*E* − 5
73	1.4997263039128846*E* − 5
74	1.5193999176213864*E* − 5
75	1.544098134602122*E* − 5
76	1.57116180097447*E* − 5
77	1.5950812116676206*E* − 5
78	1.6008865129767365*E* − 5
79	1.6154963269190848*E* − 5
80	1.6559218782739425*E* − 5
81	1.6752087063271413*E* − 5
82	1.7033959952437373*E* − 5
83	1.728771469799143*E* − 5
84	1.7564517921074768*E* − 5
85	1.7999075641112577*E* − 5
86	1.8132849402923305*E* − 5
87	1.8505092071315046*E* − 5
88	1.8708573514251417*E* − 5
89	1.8257784663164733*E* − 5
90	1.876741789470096*E* − 5
91	1.920441883415449*E* − 5
92	1.9082305553032882*E* − 5
93	1.7957378257703227*E* − 5
94	1.7963241034075204*E* − 5
95	1.836522209620149*E* − 5
96	1.8497275566932963*E* − 5
97	1.8523003590569908*E* − 5
98	1.7409426007853883*E* − 5
99	1.738247641932898*E* − 5
100	1.6890004735804907*E* − 5

**Table 3:** Percentual improvement of MSE of our hybrid model compared to the standard neural network.

Neurons	Backpropagation	*K*-means + backpropagation	Genetic algorithm
3	40,022573	21,499478	24,751698
4	25,727704	24,540767	22,364582
5	43,770397	23,404426	20,087043
6	43,321970	25,178633	32,157091
7	49,123352	20,517200	39,466626
8	69,499268	21,294707	15,976094
9	73,609061	21,175933	40,001306
10	80,630012	22,327521	18,321433

**Table 4:** Predictive comparison of tested models, best configurations (ex-post).

Model	Regressor(s)	Weights adaptation	MSE^*∗*1^	sd^*∗*2^
RBF	Autoregressive (1)	Backpropagation	0.0000183763	0.0000028765
		*K*-means + backpropagation	0.0000168649	0.0000002319
		Genetic algorithm	0.0000176860	0.0000006219

RBF-SMA	Autoregressive (1)	Back-Propagation	0.0000136485	0.0000005710
		*K*-means + backpropagation	0.0000130549	0.0000003013
		Genetic algorithm	0.0000136146	0.0000003816

AR(0)-EGARCH(1,1,1)	Conditional variance (1)	Marquardt	0.0000170651	—
		Berndt-Hall-Hall-Hausman	0.0000170651	—

^*∗*1^: mean squared error; ^*∗*2^: standard deviation.

**(a):** 

Test	Original series [*p* value]		1st differences (returns) [*p* value]	
Augmented Dickey-Fuller	(I)	−1.017396	(I)	−30.61353
		[0.2781]		[0.0000]
	(II)	−1.848666	(II)	−30.61866
		[0.3569]		[0.0000]
	(III)	−2.454401	(III)	−30.60792
		[0.3510]		[0.0000]

Phillips-Perron	(I)	−1.077154	(I)	−30.66946
		[0.2550]		[0.0000]
	(II)	−1.794202	(II)	−30.68702
		[0.3836]		[0.0000]
	(III)	−2.415434	(III)	−30.67642
		[0.3712]		[0.0000]

**(b):** 

Test	Window	Spectral estimation method							
		Bartlett kernel				Quadratic spectral kernel			
		Original series		Returns		Original series		Returns	
		(II)	(III)	(II)	(III)	(II)	(III)	(II)	(III)
KPSS *H*_0_: stationary series	Newey-West	2.735123 (0.463)	0.668131 (0.146)	0.074401 (0.463)	0.026211 (0.146)	4.846899 (0.463)	1.158977 (0.146)	0.072329 (0.463)	0.025495 (0.146)
	Andrews	0.380551 (0.463)	0.150720 (0.146)	0.065257 (0.463)	0.022956 (0.146)	0.306275 (0.463)	0.132197 (0.146)	0.065106 (0.463)	0.022906 (0.146)

Elliot-Rothenberg-Stock *H*_0_: unit root	Newey-West	30.18086 (3.26)	8.632243 (5.62)	0.358755 (3.26)	0.438046 (5.62)	29.71523 (3.26)	8.977311 (5.62)	0.352926 (3.26)	0.431096 (5.62)
	Andrews	27.06265 (3.26)	8.375229 (5.62)	0.323259 (3.26)	0.394417 (5.62)	27.29972 (3.26)	8.378941 (5.62)	0.323530 (3.26)	0.394799 (5.62)

(I): model without constant and deterministic trend (5%).

(II): model with constant and without deterministic trend (5%).

(III): model with constant and deterministic trend (5%).

**Table 6:** Normality tests on distribution of residuals and other main characteristics.

Skewness	Kurtosis	J.B.	A.D.	ARCH-LM statistic
0.168931	5.518599	245.1157	6.422445	139.4994
		[0.0000]	[0.0000]	[0.0000]

J.B.: Jarque-Bera statistic and A.D.: Anderson-Darling statistic.

**Table 7:** BDS test results on the series of AR(0) residuals.

*m*	Fraction of pairs		sd	
	BDS statistic	*z*-statistic [*p* value]	BDS statistic	*z*-statistic [*p* value]
2	0.010940	3.729713 [0.0000]	0.007757	3.545123 [0.0000]
3	0.028568	6.150556 [0.0000]	0.013889	6.456223 [0.0000]
4	0.044991	8.162869 [0.0000]	0.014137	8.905923 [0.0000]
5	0.055748	9.738419 [0.0000]	0.011450	11.16068 [0.0000]
6	0.062941	11.44079 [0.0000]	0.008584	13.98217 [0.0000]
7	0.066187	13.17452 [0.0000]	0.006155	17.62420 [0.0000]
8	0.066580	15.04687 [0.0000]	0.004189	21.85108 [0.0000]
9	0.065576	17.28602 [0.0000]	0.002799	27.30739 [0.0000]
10	0.062153	19.51334 [0.0000]	0.001781	33.16601 [0.0000]

The BDS statistic was computed by two methods, with *ε* = 0.7.

**Table 8:** Evaluation characteristics of tested models.

Model	Error distribution	Akaike	Schwarz	Log-likelihood
ARCH(5)	Gaussian	−6.946032	−6.909037	317.917
	Student	−6.966257	−6.923978	3181.130
	GED	−6.967443	−6.925164	3181.670

ARCH(7)	Gaussian	−6.970504	−6.922940	3184.065
	Student	−6.984941	−6.932092	3191.641
	GED	−6.985553	−6.932704	3191.919

GARCH(1,1)	Gaussian	−7.029560	−7.008420	3205.964
	Student	−7.032833	−7.006409	3208.456
	GED	−7.034504	−7.008779	3209.216

EGARCH(1,1,1)	Gaussian	−7.025497	−6.999073	3205.114
	Student	−7.028507	−6.996797	3207.485
	GED	−7.030426	−6.998717	3208.359

PGARCH(1,1,1)	Gaussian	−7.026622	−6.994912	3206.626
	Student	−7.029612	−6.992618	3208.988
	GED	−7.031268	−6.994274	3209.743

TGARCH(1,1,1)	Gaussian	−7.028705	−7.002281	3206.575
	Student	−7.031598	−6.999888	3208.893
	GED	−7.033244	−7.001535	3209.643

GED: generalized error.

**Table 9:** Comparison of predictive qualities (out-of-sample predictions, 1-day horizon).

Model	Error distribution	MSE	MAPE
AR(0)-ARCH(5)	Gaussian	0.00001709	0.319356
	Student	0.00001720	0.320744
	GED	0.00001718	0.320459

AR(0)-ARCH(7)	Gaussian	0.00001708	0.319096
	Student	0.00001717	0.320443
	GED	0.00001714	0.320122

AR(0)-GARCH(1,1)	Gaussian	0.00001709	0.319374
	Student	0.00001715	0.320223
	GED	0.00001714	0.320117

AR(0)-EGARCH(1,1,1)	Gaussian	0.00001706	0.318886
	Student	0.00001714	0.320108
	GED	0.00001711	0.319692

AR(0)-PGARCH(1,1,1)	Gaussian	0.00001706	0.318916
	Student	0.00001711	0.319660
	GED	0.00001712	0.319719

AR(0)-TGARCH(1,1,1)	Gaussian	0.00001706	0.318897
	Student	0.00001712	0.319767
	GED	0.00001712	0.319699

## Appendix

### Box-Jenkins Statistical Modeling

The empirical Box-Jenkins analysis [37] focuses on the original and differentiated series of daily observations of USD/CAD currency pair covering a historical period from October 31, 2008, to October 31, 2012. The reason for this particular study was to perform a comparison between our tested model and the standard statistical model. The data, as stated above, was downloaded from the following website: http://www.global-view.com/forex-trading-tools/forex-history/, and the statistical characteristics are in Figure 6.

As stated in the previous part of this paper, data was divided into two parts—the training part and validation part. As the validation part was used for model checking, we only used observations from training set for statistical modeling. For statistical modeling which included model identification, model quantification, and model validation, the Eviews software was used. Some of the advantages of this software include simplicity, user friendliness, or detailed outputs. In addition to that, it also has various versions of GARCH model implemented.

Unit root tests results [39–42] presented in Table 5 show that this series is not stationary as it is characterized by a unit root. In order to stationarize the series, it was differentiated. As seen from Table 5, unit root tests confirmed that the differentiated series was stationary which is a necessary condition in Box-Jenkins modeling.

By analyzing autocorrelation (ACF) and partial autocorrelation function (PACF) of the differentiated series of USD/CAD (see Figure 8), there were no significant correlation coefficients, so one could deduce that first differences of the original series formed a white noise process. In that case, the original series would have formed random walk process (RWP) as RWP was *I*(1) process. Assuming the returns of the original series formed a random walk process, we selected AR(0) as the basic level model. Ljung-Box *Q*-statistics confirmed this assumption and the applicability of AR(0) process as the correlations were statistically not significant.

However, the assumption of normality of residuals of AR(0) returns was rejected (see Table 6). Moreover, the observed asymmetry might have indicated the presence of nonlinearities in the evolution process of returns. This nonlinearity was confirmed by graphical quantiles comparison (versus normal distribution) and a scatter plot of the series which did not appear to be in the form of a regular ellipsoid (see Figures 9 and 10). In addition, BDS test rejected the random walk hypothesis (see Table 7) as the BDS statistic is greater than the critical value at 5%. Therefore, other tests had to be performed in order to correctly model this series.

We noted that the residuals (Figure 11) were not characterized by a Gaussian distribution (see Table 6). The asymmetry might have indicated nonlinearities in the residuals. When looking at the graph of residuals (Figure 11) one could observe that the variability of these residuals could be caused by a nonconstant variance. Residual with small value follows another residual with a small value. On the other hand, residual with a large value usually follows a residual with another large value. However, this was not typical for a white noise process. This assumption leads us to think about stochastic model for volatility. The suitability for using stochastic volatility model was also accepted by performing heteroskedasticity test. ARCH test (see Table 6) confirmed that the series was heteroskedastic since the null hypothesis of homoscedasticity was rejected at 5% and so the residuals were characterized by the presence of ARCH effect which is quite a frequent phenomenon at financial time series. Therefore, we applied a stochastic volatility model into the basic model.

We estimated several models of ARCH [43] and GARCH [1], respectively. We estimated several stochastic models; except for the basic GARCH model we estimated GARCH extensions too. For modelling the ARCH model we used the information from the correlogram of squared residuals (Figure 12). For each model we calculated Akaike [44], Schwarz [45] information criteria, and log-likelihood function. It is important to remember that the estimation of different models was only based on 912 in-sample observations, in order to make ex-ante predictions with remaining 132 observations. We used Marquardt optimization procedure for finding the optimal values of GARCH parameters; initial values of parameters were counted using Ordinary Least Squares (OLS) method and then these values by iterative process consisted of 500 iterations. Convergence rate was set to 0.0001.

In view of Table 8, we find that the information criteria are minimum for GARCH(1,1) model with GED error distribution. It showed that GARCH(1,1) is very well applied for this type of time series as well as other financial time series. Regarding the fact that we applied the models on exchange rate data, it was no surprise that the asymmetrical effect was not present. The residuals of the models were characterized by the absence of conditional heteroskedasticity: the ARCH-LM statistics are strictly less than the critical value at 5%. In addition, the standardized residuals tested with Ljung-Box *Q* test confirmed that there were no significant coefficients in residuals of these models.  Figure 13 states these results for GARCH(1,1) GED model.

To compare the forecasting performance of the tested models two criteria were used: the mean squared error (MSE) and the mean absolute percentage error (MAPE). We primarily tested the forecasting accuracy on the validation set, that is, out-of-sample predictions, and we used one-step-ahead predictions.

The best results for out-of-sample prediction were achieved with EGARCH(1,1, 1) model with Gaussian error distribution (Table 9). Therefore, this model will be later compared with the neural network models and our suggested model. This volatility is defined as follows:

(A.1) log⁡ht=−0.172109+0.117148εt−1ht+0.037398εt−1ht+0.992135log⁡ht−1.
